# Regulated Expression of *lpxC* Allows for Reduction of Endotoxicity in *Bordetella pertussis*

**DOI:** 10.3390/ijms23148027

**Published:** 2022-07-21

**Authors:** Jesús Pérez-Ortega, Ria van Boxtel, Eline F. de Jonge, Jan Tommassen

**Affiliations:** 1 Section Molecular Microbiology, Department of Biology, Faculty of Science, Utrecht University, 3584 CH Utrecht, The Netherlands; j.perezortega@uu.nl (J.P.-O.); h.a.m.tommassen@uu.nl (R.v.B.); e.f.dejonge@uu.nl (E.F.d.J.); 2 Institute of Biomembranes, Utrecht University, 3584 CH Utrecht, The Netherlands

**Keywords:** lipopolysaccharide, *lpxC*, TLR4, outer-membrane vesicles, *Bordetella pertussis*, vaccine

## Abstract

The Gram-negative bacterium *Bordetella pertussis* is the causative agent of a respiratory infection known as whooping cough. Previously developed whole-cell pertussis vaccines were effective, but appeared to be too reactogenic mainly due to the presence of lipopolysaccharide (LPS, also known as endotoxin) in the outer membrane (OM). Here, we investigated the possibility of reducing endotoxicity by modulating the LPS levels. The promoter of the *lpxC* gene, which encodes the first committed enzyme in LPS biosynthesis, was replaced by an isopropyl β-D-1-thiogalactopyranoside (IPTG)-inducible promoter. The IPTG was essential for growth, even when the construct was moved into a strain that should allow for the replacement of LPS in the outer leaflet of the OM with phospholipids by defective phospholipid transporter Mla and OM phospholipase A. LpxC depletion in the absence of IPTG resulted in morphological changes of the cells and in overproduction of outer-membrane vesicles (OMVs). The reduced amounts of LPS in whole-cell preparations and in isolated OMVs of LpxC-depleted cells resulted in lower activation of Toll-like receptor 4 in HEK-Blue reporter cells. We suggest that, besides lipid A engineering, also a reduction in LPS synthesis is an attractive strategy for the production of either whole-cell- or OMV-based vaccines, with reduced reactogenicity for *B. pertussis* and other Gram-negative bacteria.

## 1. Introduction

The cell envelope of Gram-negative bacteria consists of an inner membrane (IM) and an outer membrane (OM), separated by the periplasm where the peptidoglycan sacculus is located. While the IM is a bilayer of phospholipids (PLs), the OM shows an asymmetric organization with the PLs and lipopolysaccharides (LPS, also known as endotoxin) located in the inner and the outer leaflet, respectively [1]. The LPS consists of three moieties, i.e., lipid A, a core oligosaccharide, and a polysaccharide known as O-antigen [2]. In some bacteria, including *Bordetella pertussis*, an O-antigen is lacking, and their LPS is also referred to as lipooligosaccharide, or LOS. The LPS, or at least its lipid A moiety, appears essential for the viability of many Gram-negative bacteria, including *Escherichia coli*; however, three species, *Neisseria meningitidis* [3], *Moraxella catarrhalis* [4], and *Acinetobacter baumannii* [5], which all synthesize LPS without the O-antigen, have been reported to survive in its absence.

LPS, particularly its lipid A moiety, is responsible for the endotoxicity associated with infections by Gram-negative bacteria. This molecule is recognized by a receptor on the innate immune cells consisting of the Toll-like receptor 4 (TLR4) and myeloid differentiation factor 2 (MD-2), which triggers the production of pro-inflammatory cytokines, such as TNFα and IL-1β [2]. The endotoxic reaction elicited by the LPS is one of the main reasons for the adverse reactions evoked by whole-cell vaccines against various Gram-negative bacteria, including *B. pertussis*. This pathogen, which is responsible for the human respiratory disease known as whooping cough or pertussis, was brought under control by the worldwide introduction of whole-cell pertussis vaccines (wP) around the 1940s [6]. However, because of the reactogenicity of wP, mainly caused by its endotoxin content, acellular (aP) replacement vaccines were developed to counteract the aversion that had risen against the use of wP. These aP vaccines, which consist of one to five purified antigens, were introduced in the 1990s and proved to be effective and less reactogenic [6]. Nevertheless, during the past few decades, the number of pertussis cases has been rising in the countries using aP [7]. The apparently lower efficacy of the aP vaccines is related to the lack of protection against mucosal colonization, the fast waning of the vaccine-induced immune protection, and the emergence of bacterial variants with mutations in the genes for vaccine antigens [7]. The differences in the immune response upon vaccination with wP versus aP seem to be one of the main reasons for the difference in efficacy. While wP elicits particularly strong T-helper (Th)1 and, to a lesser extent, Th17 responses, aP elicits a mixed Th2 and Th17 reaction. A Th1 response appears to be crucial for protection against pertussis colonization [7,8]. These data point towards the need for a new Th1- and Th17-response-inducing pertussis vaccine with low reactogenicity, preferably one with an abundant number of bacterial antigens to avoid the vaccine-induced selection of escape mutants.

A solution could be the development of a novel whole-cell vaccine, but a prerequisite for this option is that its endotoxicity should be diminished. Another approach gaining ground in the protection against Gram-negative bacteria is the use of vaccines based on OM vesicles (OMVs). OMVs are blebs derived from the OM that are spontaneously released from the bacterial surface in multiple environmental conditions [9]. These vesicles present bacterial surface antigens in combination with LPS and have robust immunogenic capacity. They have been shown to be an effective alternative in vaccine development against Gram-negative pathogens, in particular *N. meningitidis* [10,11]. For *B. pertussis*, immunization studies in mice with an OMV-based vaccine showed a lower production of pro-inflammatory cytokines compared to wP immunization. In addition, the OMV vaccine induced an immune protection similar to wP, including a Th1 response, which was not elicited by aP [12]. Nevertheless, whether the reduction in endotoxicity in these OMVs is enough to produce acceptably safe vaccines for human use remains unknown.

A prevalent strategy to reduce the endotoxicity of bacterial cells, which could also be applied to OMVs, is through the genetic engineering of the lipid A structure to weaken its interaction with TLR4 [13,14], an approach that has also been applied in *B. pertussis* [15,16]. However, a reduction in the total amount of LPS might be an alternative approach to accomplish the appropriate endotoxin levels.

The amount of LPS produced in bacteria is meticulously regulated at different levels. The biosynthesis pathway of lipid A is known as the Raetz pathway [2]. The first committed reaction in this process is catalyzed by the UDP-3-*O*-acyl-*N*-acetylglucosamine deacetylase LpxC and is a crucial point for the regulation of LPS production. The cellular LpxC levels directly correlate with the LPS production and are governed, at least in some enteric bacteria, by the FtsH/LapB (also known as YciM) protease complex, which degrades LpxC [17,18]. In addition, YejM (also known as PbgA) prevents the FtsH/LapB-mediated LpxC proteolysis and, therefore, stabilizes the LpxC levels [19,20,21,22,23]. However, the LpxC regulatory systems might work differently in other bacteria, as seems to be the case for *Pseudomonas aeruginosa* [17,24] and *N. meningitidis* [17,25], for example, and therefore, the mechanism remains unknown for many other species, including *B. pertussis*. Additionally, the LpxC levels can be indirectly controlled in the OM by the OM phospholipase A (OMPLA), encoded by the *pldA* gene, which degrades the mislocalized PLs that may appear in the outer leaflet of the OM when the LPS levels are deficient. The OMPLA, then, releases fatty acids from these PLs, which form a signal to inhibit the LpxC proteolysis and, thereby, increase the LPS production [26]. Consistently, the inactivation of the maintenance of lipid asymmetry (Mla) system, which mediates the retrograde transport of aberrantly localized PLs from the outer leaflet of the OM to the IM, prompts the accumulation of mislocalized PLs, which activates OMPLA and eventually stimulates LPS production [27]. Accordingly, the combined inactivation of OMPLA and the Mla system in *B. pertussis* results in an accumulation of PLs in the outer leaflet of the OM [28]. In this study, we investigated whether it is possible to reduce the amount of LPS in *B. pertussis* whole-cell preparations and OMVs by regulating the *lpxC* expression.

## 2. Results

### 2.1. Regulated Expression of lpxC and Its Implication on Growth

Since *lpxC* (locus tag BP3017) was suggested to be an essential gene in *B. pertussis* [29,30], we set out to regulate the expression of the *lpxC* gene and, consequently, the production of LPS in the cells. To this end, plasmid pUCK-*lpxC* was constructed (Figure S1, Supplementary Materials). This plasmid, which doesn’t replicate in *Bordetella*, contains an *lpxC* allele that is truncated because of a premature stop codon. It can integrate into the bacterial chromosome via homologous recombination into the *lpxC* gene. As a result of integration, the chromosomal gene is disrupted and an additional, IPTG-regulated, intact copy of the gene is created, which is controlled by the dual *tac*-*lac* promoter included in the plasmid (Figure S1, Supplementary Materials). The presence of an ampicillin-resistance marker in the plasmid allowed for the selection of recombinants containing the construct integrated into the chromosome.

**Figure 1:**
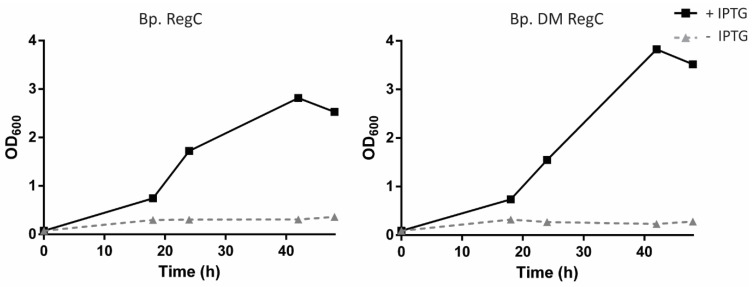
Growth of *B. pertussis* strains with regulatable *lpxC* in the presence or absence of 1 mM IPTG. Growth curves of Bp. RegC (**left panel**) and Bp. DM RegC (**right panel**) were measured at OD_600_ for 48 h in the presence or absence of IPTG. Each graph shows values of a single experiment.

After obtaining a *B. pertussis* strain containing the *lpxC* regulatory construct (Bp. RegC), we tested whether it could grow in the absence of IPTG and, thus, in the absence of LPS production. After growing strain the Bp. RegC on a Bordet–Gengou agar plate, supplemented with sheep blood (BG-blood), ampicillin, and IPTG, the cells were scraped from the plate and resuspended in a liquid medium with ampicillin and either with or without the inducer. Without IPTG, a slight increase in the optical density at 600 nm (OD_600_) of the culture was observed in the first hours, after which the OD_600_ remained static for the next 48 h (Figure 1, left panel), indicating the inability of the bacteria to grow in the absence of *lpxC* expression.

In the absence of LPS synthesis, the PLs are presumed to reach the outer leaflet of the OM to replace the LPS. However, such mislocalized PLs are degraded by activated OMPLA, or transported back to the inner membrane by the Mla system. Such activities would prevent the formation of a stable phospholipid bilayer in the OM. Accordingly, although *A. baumannii* is viable in the absence of the LPS synthesis, the growth of such mutants is severely restricted but can be restored by the inactivation of OMPLA and the Mla system [31,32]. Thus, we decided to test whether we could rescue a *B. pertussis* strain lacking LPS in the same way. To that end, the *lpxC*-regulatory construct was introduced into a *B. pertussis* strain in which OMPLA and the Mla system were inactivated by *pldA* and *mlaF* mutations, respectively. However, these modifications didn’t allow for the growth of the resulting strain, Bp. DM RegC, in the absence of IPTG (Figure 1, right panel).

### 2.2. LPS-Depletion Protocol

**Figure 2:**
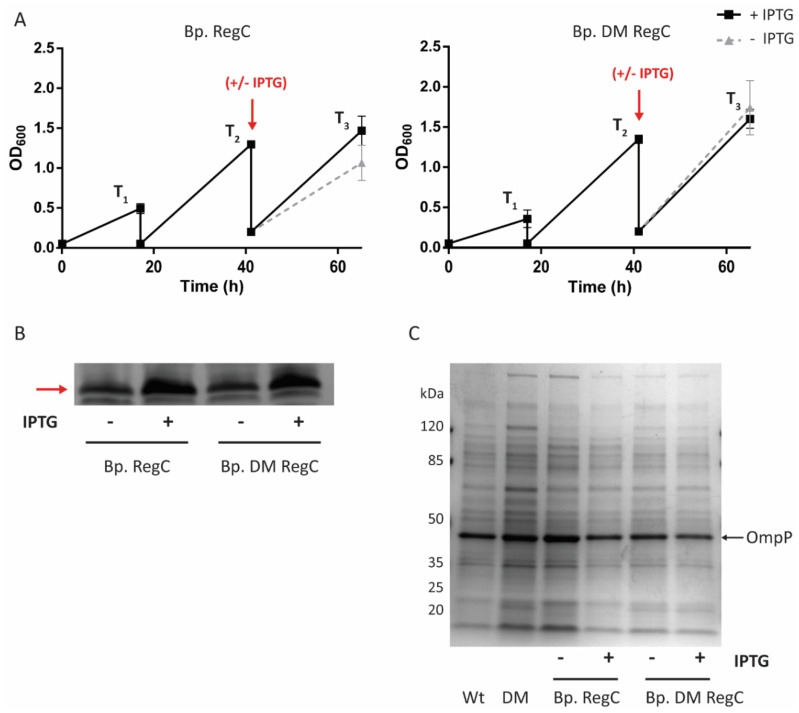
Growth of *B. pertussis* strains with regulatable *lpxC* during LPS-depletion conditions and characterization of the cell content. (**A**) Growth curves of Bp. RegC (left panel) and Bp. DM RegC (right panel) were measured at OD_600_ following the LPS-depletion growth protocol discussed in the text. At T_2_, bacterial cultures were washed and grown further either without IPTG (grey dashed line) or with 1 mM IPTG (black solid line). Graphs show mean values with standard deviations of three independent experiments. No significant differences were found with a paired *t*-test; (**B**) Analysis of the LPS content of cells by SDS-PAGE. Samples taken at T_3_ in panel A were adjusted based on OD, analyzed by SDS-PAGE, and LPS was visualized by silver staining. Only the relevant part of the gel is shown. The band corresponding to LPS is indicated with an arrow; (**C**) The protein content of OM preparations from Bp. RegC and its wild-type parental strain (Wt) and from Bp. DM RegC and its parental strain (DM) was analyzed by SDS-PAGE. The major OM protein, porin OmpP, is indicated with an arrow at the right. Molecular-weight markers are indicated at the left.

Since the constructed mutants can apparently not grow in the absence of IPTG, we wanted to develop a protocol for the growth of Bp. RegC that allows for obtaining sufficient bacterial cells but with reduced amounts of LPS. In this protocol (Figure 2A), Bp. RegC cells were grown on BG-blood plates containing IPTG and resuspended in liquid medium supplemented with IPTG. After 17 h of growth (T_1_), the cells were diluted into fresh medium with IPTG to an OD_600_ of 0.05. After 24 h of growth of this preculture (T_2_), the cells were washed twice with medium without IPTG by centrifugation for 3 min at 7000× *g*, adjusted to an OD_600_ of 0.2, and grown either with or without IPTG for another 24 h (T_3_). In the absence of IPTG, an apparent, but statistically not significant, reduction in the growth was observed (Figure 2A, left panel). Interestingly, such a reduction in the growth was not observed for Bp. DM RegC (Figure 2A, right panel), suggesting that the accumulation of PLs in the outer leaflet of the OM can compensate to some extent for the downregulation of *lpxC*, even though the *lpxC* expression is also essential for viability in this strain (Figure 1).

The whole cells collected at the end of the LPS-depletion protocol (T_3_) were analyzed by sodium dodecyl sulfate-polyacrylamide gel electrophoresis (SDS-PAGE). A silver staining of the gel showed reduced LPS levels in strains Bp. RegC and Bp. DM RegC grown in the absence of IPTG compared to the cells grown with IPTG (Figure 2B). The protein profiles of the isolated OMs of the parental strains and their *lpxC*-regulated derivatives were comparable, even after growth in the LPS-depletion conditions (Figure 2C).

### 2.3. LPS Depletion Affects Cell Shape

**Figure 3:**
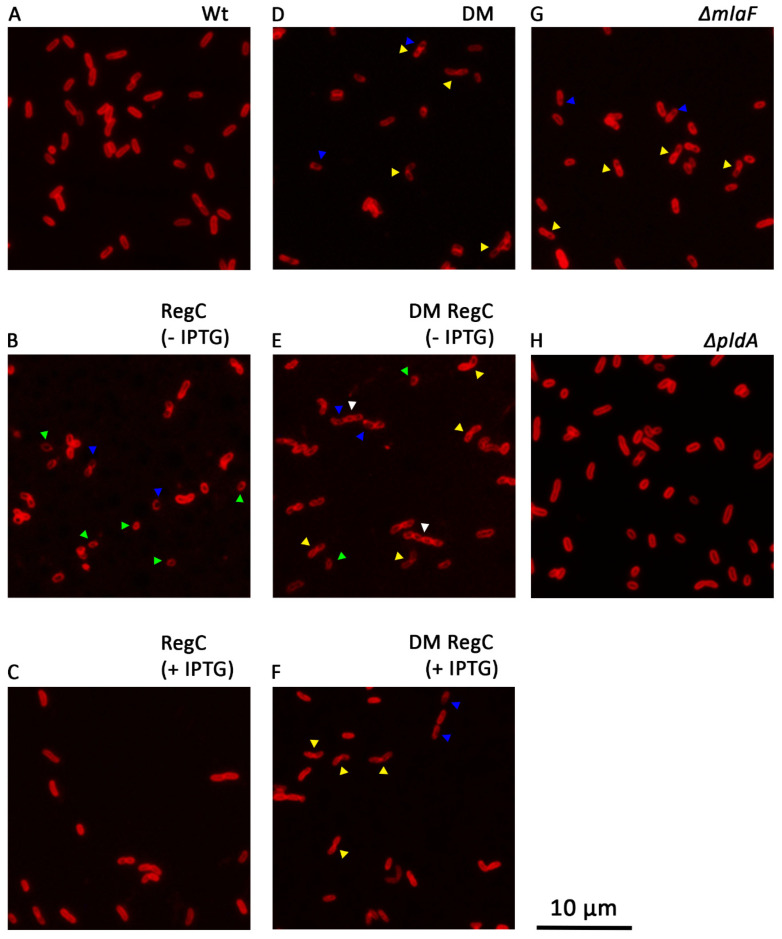
Morphology of *B. pertussis* cells visualized by fluorescence microscopy. Representative captions of the wild-type (Wt) (**A**), Bp. RegC (**B**,**C**) grown in absence (−) or presence (+) of IPTG, DM mutant (**D**), Bp. DM RegC (**E**,**F**) grown in absence (−) or presence (+) of IPTG, and the *mlaF* (**G**) and *pldA* (**H**) mutant strains are included. Wt and the Δ*mlaF*, Δ*pldA*, and DM mutant strains were grown in liquid medium for 17 h, and strains Bp. RegC and Bp. DM RegC were grown with 1 mM IPTG or without IPTG following the LPS-depletion protocol and analyzed at T_3_ as defined in Figure 2A. The cells were stained with the fluorescent dye FM4-64. Scale bar represents 10 µm. Examples of cells showing a shorter and more rounded shape than the wild-type cells (green), uneven dye distribution (blue), or appearing in pairs (yellow) or short chains (white) are indicated with colored arrowheads. Enlargements of details of these figures are shown in Figure S2 (Supplementary Materials).

We next wanted to study the effect of the LPS depletion on cell morphology. To this end, the bacteria were stained with the fluorescent membrane dye FM4-64 and examined by fluorescence microscopy (Figure 3; details of the frames shown in Figure 3 are shown enlarged in Figure S2, Supplementary Materials). Whereas the wild-type strain showed, as expected, short rods (Figure 3A and Figure S2A, Supplementary Materials), the Bp. RegC cells appeared shorter and more rounded than the wild type when the bacteria were grown in the absence of IPTG (examples indicated with green arrowheads in Figure 3B and Figure S2B, Supplementary Materials), and this phenotype was suppressed after growth of the strain in the presence of IPTG (Figure 3C and Figure S2C, Supplementary Materials). In addition, we observed an uneven distribution of the dye on the surface of the LPS-depleted mutant cells (blue arrowheads in Figure 3B and Figure S2B, Supplementary Materials), which suggests alterations in the membrane composition and/or integrity.

While the cells observed in the wild type were mostly singular cells (Figure 3A and Figure S2A, Supplementary Materials), the *mlaF pldA* double mutant Bp. DM more frequently showed pairs of cells (examples indicated by yellow arrowheads in Figure 3D and Figure S2D, Supplementary Materials) suggesting a defect in the cell division. We hypothesized that this defect is due to the inactivation of either OMPLA or the Mla system in Bp. DM. Interestingly, many pairs of the cells were also detected in an *mlaF* single mutant (yellow arrowheads in Figure 3G and Figure S2G, Supplementary Materials) but not in a *pldA* single mutant (Figure 3H and Figure S2H, Supplementary Materials). These results indicate a novel role of the Mla system in cell division. When Bp. DM RegC was deprived of *lpxC* expression, we observed many short chains of cells (white arrowheads in Figure 3E and Figure S2E, Supplementary Materials), which suggests that the defect in the cell division is exacerbated by the lack of LPS. The cell chaining was reduced again when this mutant was grown with IPTG, and many pairs of cells were observed under these conditions (Figure 3F and Figure S2F, Supplementary Materials).

### 2.4. LPS Depletion Affects OMV Production

**Figure 4:**
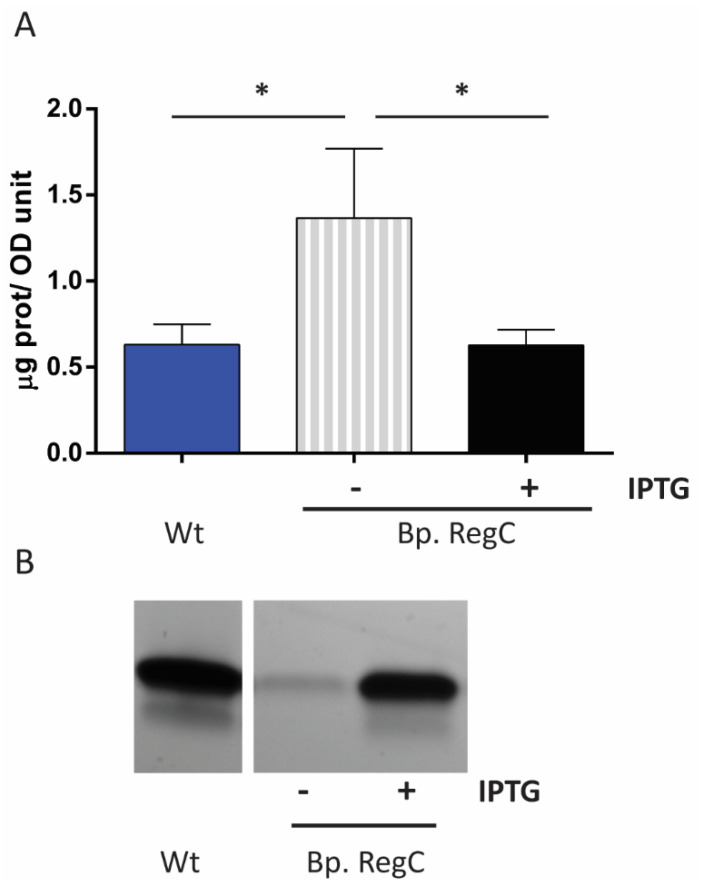
Influence of LPS depletion on OMV production. OMVs were isolated from the supernatant of the same volumes of cultures of the wild-type strain (Wt) and of the Bp. RegC strain obtained at T_3_ as defined in Figure 2A following the LPS-depletion protocol with or without IPTG. (**A**) OMVs were quantified based on protein content using a Lowry assay. Protein content is depicted as the amount of protein in the OMV fractions per liter of bacterial culture per OD_600_ unit. Values shown are means and standard deviations from three independent experiments, and significant differences are indicated with asterisks (*p* ≤ 0.05); (**B**) LPS in the isolated OMVs was analyzed by SDS-PAGE after adjusting the samples to similar protein content, and the LPS was visualized in the gel by silver staining.

To determine whether the OMV production was affected by the reduction in the LPS content, we isolated the OMVs from the liquid cultures grown following the LPS-depletion protocol (at T_3_ in Figure 2A), and the amounts of the isolated OMVs were quantified on the basis of protein content. As shown in Figure 4A, the OMV production was significantly enhanced (~two-fold increase) relative to the wild type when Bp. RegC was grown in the absence of IPTG, and this overproduction was suppressed when the strain was grown in the presence of IPTG. To examine the LPS content of the OMVs, the OMV preparations were adjusted to protein content and analyzed by SDS-PAGE. Remarkably, the intensity of the LPS band was much lower in the OMV preparations from the mutant cells lacking *lpxC* expression than in those from the cells grown with IPTG or from the parental strain (Figure 4B), and the difference in the LPS content in the OMVs appeared considerably larger than in the whole-cell lysates of the strains (Figure 2B).

### 2.5. LPS Depletion Reduces TLR4 Activation by Whole Cells or OMVs

**Figure 5:**
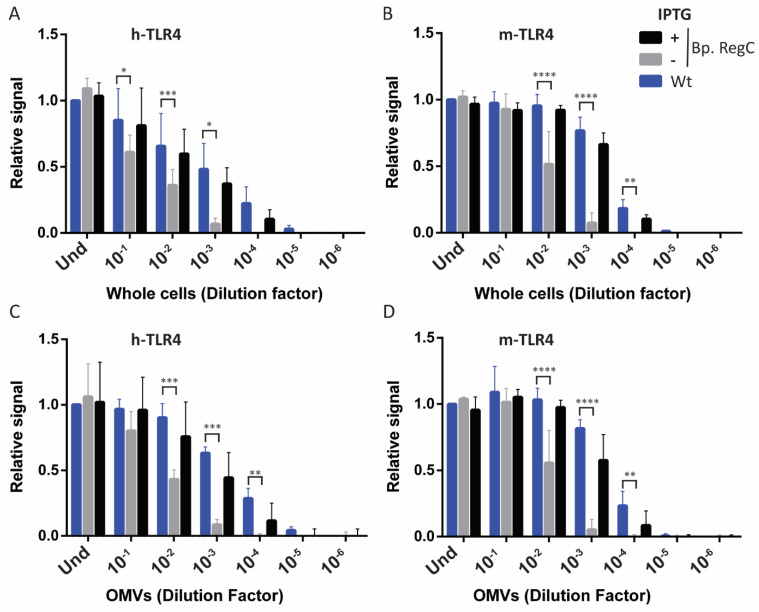
TLR4 activation by whole-cell and OMV preparations. HEK-Blue cells expressing either h-TLR4 (**A**,**C**) or m-TLR4 (**B**,**D**) were incubated for 17 h with 10-fold serial dilutions of heat-inactivated whole cells (the OD_600_ of the undiluted cell suspensions (Und) was 0.15) (**A**,**B**) or isolated OMVs (the protein concentration in the undiluted preparations (Und) was 1 µg/mL) (**C**,**D**). Diagrams show means and standard deviations of relative SEAP activity calculated as the ratio between the signal measured for each dilution of each strain and the signal measured for Und. Wt (parental strain). Three independent experiments were performed in duplicate (whole cells) or singularly (OMVs). Statistical comparisons showed significant differences relative to the wild type for Bp. RegC grown in absence of IPTG but not for Bp. RegC grown with IPTG. Dilutions with statistically different results are indicated with asterisks (*, *p* ≤ 0.05; **, *p* ≤ 0.01; ***, *p* ≤ 0.001; ****, *p* ≤ 0.0001).

To determine if the reduced LPS content in the cells and OMVs of strain Bp. RegC grown in the absence of IPTG reduces TLR4 signaling, we tested TLR4 activation in the HEK-Blue reporter cells expressing either human or murine TLR4 (h- and m-TLR4, respectively) after their stimulation with whole-cell or OMV preparations (Figure 5). The whole-cell preparations of the parental strain and of strain Bp. RegC induced with IPTG showed similar TLR4 activation, while the preparations of strain Bp. RegC grown in the absence of IPTG showed significantly reduced h- and m-TLR4 stimulation, with 10- to 100-fold higher cell concentrations being required for a similar activation of the receptors (Figure 5A,B). Similarly, the OMV preparations were tested. The OMV preparations used were adjusted to a similar protein content. Following the trend observed for the whole-cell preparations, the OMVs obtained from Bp. RegC after growth in the absence of IPTG showed a drastically reduced activation of both h-TLR4 (Figure 5C) and m-TLR4 (Figure 5D).

## 3. Discussion

Immunization with wP vaccines was discontinued in the last 30 years in industrialized countries because of their high reactogenicity, which was mainly due to the presence of LPS in these vaccines. They were replaced by aP vaccines, which proved to be safer, but also seemed to be less effective, considering the resurgence of pertussis in the last few decades [7,8]. Thus, new pertussis vaccines are needed. Such new vaccines should preferably be based on whole cells because of the multitude of antigens they contain and the Th1/Th17-type response that such vaccines induce. However, a prerequisite is that the reactogenicity is reduced relative to the original wP vaccines.

An alternative approach is the use of OMV-based vaccines, which also contain a multitude of relevant antigens and have been proven to be effective for another Gram-negative bacterium, i.e., *N. meningitidis* [33]. The OMV-based vaccines that have been licensed to date consist of detergent-extracted OMVs, a procedure that reduces the LPS content. However, this treatment can also reduce the content of relevant surface antigens [33]. Preliminary in vivo studies indicated that the OMV-based vaccines can also be used for *B. pertussis* [12]. In that study, it was demonstrated that these vaccines can provide comparable protection in mice to whole-cell formulations, but they showed lower endotoxicity, which makes them a safer alternative. However, the reason for the lower reactogenicity of these OMV preparations is unknown, and it remains to be established whether reactogenicity is sufficiently reduced for application in humans. Hence, additional measures to reduce endotoxicity may be required.

A commonly applied method to reduce the endotoxicity of whole-cell preparations is lipid A engineering [13,14,34,35]. The endotoxicity of lipid A is determined by the number and the length of its fatty acyl chains, as well as by its phosphate groups [36,37], which can all be modified by genetic engineering. In *B. pertussis*, the genetically induced modifications of lipid A have successfully been applied to reduce the endotoxicity. This was achieved by using different approaches, i.e., by shortening the length [16], or reducing the number of acyl chains [15]. The latter approach, however, which was achieved by the expression of the lipid A deacylase PagL in the cells, resulted in less toxic LPS, but the whole-cell preparations showed increased endotoxicity, probably as a result of an increased release of LPS from the bacterial surface [15].

Here, we present an alternative strategy to reduce endotoxicity, i.e., by genetically interfering with the LPS synthesis, which was achieved by controlling *lpxC* expression. This strategy can be applied to reduce the endotoxicity of both whole-cell and OMV preparations, and is potentially also applicable in the development of vaccines against other bacteria. Previously, whole-cell formulations with a reduced LPS content have also been developed [38,39]. However, this reduction was commonly achieved by the treatment of whole cells with organic solvents, which can cause the loss of other OM components, e.g., hydrophobic (lipo)proteins [40]. With our strategy, we reduced the amount of LPS, while avoiding harsh treatments that could cause the loss of surface-exposed antigens that are relevant for optimal immune protection.

Although LPS is essential for the viability of many Gram-negative bacteria, some of them, including *A. baumannii*, can survive without it [3,4,5]. Such mutant strains of *A. baumannii* grow poorly, but growth could be considerably improved by the inactivation of OMPLA and the Mla system, which degrade or remove PLs showing up in the outer leaflet of the OM, respectively [31,32]. Our results demonstrate that the LPS synthesis is essential for the viability of *B. pertussis*. We considered the possibility that the formation of a stable bilayer of PLs in the OM in the absence of LPS synthesis is prevented by the activity of OMPLA and the Mla system. The combined inactivation of the OMPLA and the Mla system was previously demonstrated to allow for the accumulation of PLs in the outer leaflet of the OM of *B. pertussis* [28]. However, the combined inactivation of the *pldA* and *mlaF* genes appeared to be ineffective or, at least, insufficient to sustain the growth of *B. pertussis* in the absence of *lpxC* expression. Thus, we did not succeed in constructing a strain totally devoid of LPS. However, besides its adverse effects, LPS is also a potent adjuvant. The activation of TLR4 by LPS seems to play an important role in the induction of the appropriate cellular immune responses, i.e., Th1 and Th17 [41], and even the addition of the LPS analogs as adjuvant to an aP vaccine has been reported to be effective [42]. Our *lpxC*-regulated strain could allow for the fine-tuning of the LPS amount to the needs for a safe and effective vaccine formulation with sufficiently retained adjuvant activity.

Additionally, de Jonge et al. [28] showed that the disrupted lipid asymmetry in the OM of an *mlaF pldA* double mutant results in increased OMV production, which is in line with the results of a previous study in other species [43]. Likewise, we observed the overproduction of OMVs in the LpxC-depleted strain, probably as a consequence of the increased amounts of PLs in the outer leaflet of the OM. The LpxC depletion resulted in a stronger reduction in the LPS content in OMVs than in the whole cells (compare Figure 2B and Figure 4B), suggesting that increased blebbing serves to shed accumulated PLs from the OM and to restore the LPS/PL stoichiometry. A similar observation was made in a YejM/PbgA-depletion strain of *E. coli*, which produced OMVs highly enriched in PLs, apparently to compensate for the decreased levels of LPS in the OM [22]. The overproduction of OMVs in the LpxC-depletion strain could improve the cost efficiency of an OMV-based vaccine against *B. pertussis*.

The depletion of LPS also resulted in morphological defects as the cells became smaller and coccoid in the absence of *lpxC* expression. Interestingly, such morphological alteration was also reported for LPS-depleted *A. baumannii* [31]. Additionally, in the strain in which the Mla system and OMPLA were inactivated, chains of cells were formed, especially in the absence of *lpxC* expression (Figure 3E and Figure S2E, Supplementary Materials). Interestingly, similar chains of cells can be observed in the micrographs of an *A. baumannii* Δ*mlaA* Δ*pldA* Δ*lpxC* triple mutant strain, but not in those of a Δ*lpxC* single mutant [31]. This analogy suggests that chaining occurs as a consequence of one or a combination of these mutations, and that this phenotypic deviation is shared by different bacterial species. We suggest that the inactivation of the Mla system is responsible for this morphological change, since our *mlaF* single mutant already showed a division defect. The additional absence of *lpxC* expression may then exacerbate the division defect. Thus, these observations suggest a direct or indirect role of the Mla system in cell division.

Overall, we present a new strategy to reduce the endotoxicity of the whole-cell- or OMV-based vaccines against Gram-negative bacteria. The strain we created allows for the production of *B. pertussis* cells with a substantial reduction in the LPS content and, consequently, a reduced TLR4-stimulating activity. In addition, we did not observe evident modifications of the protein content of the OM. In the same vein, this approach could be used for the development of OMV-based vaccines, in as much as it allows for a drastic reduction in the LPS content in OMVs, while increasing the OMV production. However, further strain development may be needed for commercial vaccine manufacturing. The presence of the antibiotic-resistance cassettes and vector sequences is undesirable in a vaccine strain, and they should be removed. Furthermore, the use of an authentic inducible *Bordetella* promoter instead of the dual *tac*-*lac* promoter and a switch to a more recent *B. pertussis* isolate instead of the Tohama I derivative used in this study may be considered. Additionally, the LPS-depletion protocol will probably have to be adapted to large-scale culturing in fermenters, and the immunogenicity and reactogenicity of preparations still have to be tested in vivo. Nevertheless, our study already provides proof-of-principle for a strategy by which the LPS levels in whole-cell and OMV formulations are genetically controlled to generate novel vaccines with reduced reactogenicity.

## 4. Materials and Methods

### 4.1. Bacterial Strains and Growth Conditions

All of the bacterial strains used are described in Table S1 (Supplementary Materials). The *E. coli* strains were grown at 37 °C in lysogeny broth (LB) while shaking or on LB agar plates. The *B. pertussis* strain B213 and its derivatives were grown at 35 °C on Bordet–Gengou agar (Difco), supplemented with 15% defibrinated sheep blood (Biotrading, Mijdrecht, The Netherlands) (BG-blood). For the liquid cultures, the bacteria were scraped from BG-blood plates after three days of growth and used to inoculate Verwey medium [44] to an OD_600_ of 0.05. The bacteria were then grown at 35 °C while shaking at 175 rpm. Further details regarding the growth conditions are indicated in the Results section. When required for plasmid maintenance or strain selection, 100 µg/mL of ampicillin was included in the medium. To induce gene expression, the media were supplemented with 1 mM IPTG.

### 4.2. DNA Manipulation and Plasmid Construction

All of the plasmids and PCR primers used are listed in Tables S1 and S2 (Supplementary Materials), respectively. The regular PCR reactions were performed using DreamTaq DNA polymerase (Thermo Scientific, Waltham, MA, USA), whilst the PCR fragments generated for cloning were obtained using the Expand High Fidelity PCR system (Roche Diagnostics GmbH, Mannheim, Germany). For the purification of the PCR products, the commercial Wizard SV Gel and PCR Clean-Up System (Promega, Madison, WI, USA) was employed. The plasmids were isolated with the E.Z.N.A. Plasmid Mini Kit I (Omega Bio-Tek, Norcross, GA, USA). The PCR products and plasmids were digested with the appropriate restriction enzymes (Thermo Scientific), according to the manufacturer’s instructions, purified, and ligated using T4 DNA ligase (5 U/µL) (Thermo Scientific).

The cloning procedures were performed in *E. coli* strain DH5α by transformation, using the CaCl_2_ method. For the construction of the regulated *lpxC*-expression plasmid, a DNA fragment, containing the dual *lac* and *tac* promoters, the *lacI^q^* regulatory gene, and the erythromycin-resistance cassette from pEN11-NMB0338, was amplified by PCR using the primers pENP_ery_Fw and pENP_Rev_NdeI. The PCR product obtained and the vector pUC18 were digested with the endonucleases PstI and NdeI and, subsequently, ligated together, generating plasmid pUCK. The nucleotide sequence of the insert was validated by DNA sequencing. The primers RegLpxC-FW-NdeI and RegLpxC-RV-AatII were used to amplify an 807-bp truncated *lpxC* gene, with a premature stop codon introduced via the reverse primer. The amplicon was inserted into pUCK after digestion of the vector and PCR product with NdeI and AatII, resulting in pUCK-*lpxC*. After the validation of the insert by DNA sequencing, the plasmid pUCK-*lpxC* was introduced into *B. pertussis* by electroporation. The electrocompetent cells were prepared from cultures grown for 72 h, which were harvested by centrifugation for 10 min at 10,000× *g*, and the cells were washed twice with MilliQ water, which was precooled at 4 °C, in a volume equal to that of the discarded medium. The cells were subsequently washed in precooled 300 mM sucrose with one fourth of the original volume, resuspended in precooled 300 mM sucrose to an OD_600_ of 50, and stored in aliquots at −80 °C till further use. After thawing the electrocompetent cells, 3 μL of plasmid DNA (~1 μg) was added to 40 μL of the cell suspension in a 0.1-cm cuvette (Bio-Rad, Hercules, CA, USA), and electroporation was executed at 200 Ω, 25 μF, and 2.25 kV. The electroporated cells were mixed with prewarmed Verwey medium and incubated at 35 °C for 75 min before spreading them on BG-blood plates containing ampicillin for selection and IPTG for inducing gene expression. The integration of the construct in the genome was confirmed by PCR, using primers pENP-Sh-Fw-XbaI and Rv-LpxC-dw400.

### 4.3. OM Isolation

The bacterial cells were collected from the liquid cultures by centrifugation at 10,000× *g* for 10 min at 4 °C, resuspended in 2 mL of physiological salt solution to an OD_600_ of 7.5, and inactivated by incubation for 30 min at 56 °C. Then, the cells were harvested by centrifugation at 10,000× *g* for 10 min at 4 °C. The spheroplasts were made, as previously described [45]. Briefly, the cells were resuspended in 2 mL of 0.75 M sucrose and 10 mM Tris-HCl (pH 7.8). Then, 10 µL of 40 mg/mL lysozyme was added, followed by 4 mL of 1.5 mM EDTA (pH 7.5). The suspension was incubated for 30 min at room temperature. The spheroplasts were frozen at −80 °C, thawed, and lysed by ultrasonication. The unbroken cells and aggregates were removed by centrifugation at 10,000× *g* for 1 h at 4 °C. The supernatant was then centrifuged for 1 h at 40,000 rpm (Beckman Coulter Optima LE-80K, Type 70 Ti rotor, Brea, CA, USA) at 4 °C, and the resulting pellet was resuspended in phosphate-buffered saline (PBS).

### 4.4. OMV Isolation

The OMVs were isolated, as previously described [46] with slight modifications. Briefly, the bacterial cells from the liquid cultures were pelleted by centrifugation at 10,000× *g* for 10 min, and the supernatants were passed through a 0.45-µm pore-size filter (Sarstedt, Nümbrecht, Germany). The OMVs were pelleted by ultracentrifugation for 2 h at 40,000 rpm and 4 °C (Beckman Coulter Optima LE-80K, Type 70 Ti rotor) and resuspended in PBS. The OMVs were quantified based on protein content, using a Lowry DC protein assay (Bio-Rad), according to the manufacturer’s instructions.

### 4.5. SDS-PAGE

The whole cells, OM preparations, or isolated OMVs were mixed with the sample buffer [47], boiled for 10 min, and analyzed on 8–16% Mini-PROTEAN TGX Precast Protein Gels (Bio-Rad). After electrophoresis, the protein profiles were visualized by staining with Bradford reagent [48], and the LPS was stained with silver [49].

### 4.6. Microscopy

The bacterial cultures were fixed with 1% formaldehyde for at least 30 min at 4 °C and stained with 5 µg/mL of FM4-64 (Invitrogen, Waltham, MA, USA) for 15 min in the dark. Then, 5 µL of the stained suspension was pipetted onto 1%-agarose pads placed on microscopy slides, and cells were visualized with a Zeiss Axioskop 2 fluorescence microscope with a 100× objective (Oberkochen, Germany).

### 4.7. TLR4 Stimulation Assays

The TLR4 stimulation assays were performed, as previously described [35]. Briefly, the HEK-Blue TLR4 reporter cells (Invivogen) were incubated with serial dilutions of either whole bacterial cells that were killed by heat treatment at 56 °C for 1 h or the isolated OMVs. After 17 h of incubation at 37 °C in a 5% saturated CO_2_ atmosphere, supernatants were incubated with *p*-nitrophenyl phosphate solution for 1 h, and the absorbance at 405 nm was measured in a Biotek microplate reader (Winooski, VT, USA).

### 4.8. Statistical Analysis

All of the statistical analyses were performed using the GraphPad Prism software version 6. The TLR4 stimulation data were analyzed for statistical significance using two-way ANOVA (Dunnett’s correction for multiple comparisons), while one-way ANOVA (Tukey’s correction for multiple comparisons) was applied on OMV quantification studies.

## Data Availability

Data are contained within the article and its Supplementary Materials. Additional data supporting the findings of this study are available from the corresponding author on reasonable request.

## Supplementary Materials

The following supporting information can be downloaded at: https://www.mdpi.com/article/10.3390/ijms23148027/s1. References [50,51,52,53] are cited in the supplementary materials.
