# Supplementary material for: Regulated Expression of lpxC Allows for Reduction of Endotoxicity in Bordetella pertussis
Source: Int J Mol Sci. 2022 Jul 21;23(14):8027. doi: 10.3390/ijms23148027 (PMC9324023; doi:10.3390/ijms23148027)
Supplement: Supplementary file 1 [file ijms-23-08027-s001.zip › ijms-1756529-supplementary.pdf]

Table S1 | Bacterial strains and plasmids

Strain / plasmid	Description [a]	Reference
B. pertussis		
B213	Str^{R} derivative of strain Tohama I	[50]
B213 Δ*mlaF*	Δ*mlaF::gem* mutant of B213, Str^{R}, Gem^{R}	[28]
B213 Δ*pldA*	Δ*pldA::gem* mutant of B213, Str^{R}, Gem^{R}	[28]
Bp. DM	Δ*pldA::gem* mutant of B213 Δ*mlaF*, Str^{R}, Gem^{R}	[28]
Bp. RegC	B213 with integrated pUCK-lpxC, Str^{R}, Amp^{R}, Ery^{R}	This study
Bp. DM RegC	Bp. DM with integrated pUCK-lpxC, Str^{R}, Gem^{R}, Amp^{R}, Ery^{R}	This study
E. coli		
DH5α	F^{-}, Δ(*lacZYA-argF)U169 thi-1 hsdR17 gyrA96 recA1 endA1 supE44 relA1 phoA Φ80 dlacZΔM15*	[51]
Plasmids		
pEN11-NMB0338	pEN11 [52] harboring NMB0338, Ery^{R}, Cam^{R}	Unpublished
pUC18	Cloning vector, Amp^{R}	[53]
pUCK	pUC18 derivative harboring dual *lac* and *tac* promoter, *lacI*^{q} and Ery^{R} cassette from pEN11-NMB0338, Amp^{R}, Ery^{R}	This study
pUCK-***lpxC***	pUCK derivative harboring truncated *lpxC* gene from *B. pertussis* B213, Amp^{R}, Ery^{R}	This study

[a] Str, streptomycin; Gem, gentamicin; Amp, ampicillin; Ery, erythromycin; Cam, chloramphenicol

Table S2 | PCR primers used in this study

Primer	Sequence (5'→3') [a]	Description
pENP_ery_Fw	GCGCGCGGTACCGGTATCAACACTGCAGAA	Primers for amplification of pEN11 fragment including p*lac*, p*tac*, *lacI*^{q}, and erythromycin-resistance cassette, introducing PstI and NdeI restriction sites
pENP_Rev_NdeI	CGCGCGCATATGCAGTTCCTTGTGGTGCGGA	
RegLpxC-FW-NdeI	GCGCGCCATATGTTCCGACAGCGCAGTATTC	Primers for amplification of *B. pertussis lpxC* introducing premature stop codon and NdeI and AatII restriction sites
RegLpxC-RV-AatII	GCGCGCGACGTC**CTA**ATGGCCCGATTTGTAGGCAAC	
pENP-Sh-Fw-XbaI	GCGCGCTCTAGATGTGGAATTGTGAGCGGATA	Primers for confirmation of construct integration aligning at the promoter of the vector and the chromosomal sequence downstream the target gene.
Rv-LpxC-dw400	GAACCAGCATCTGCAGTTG	

[a] Restriction sites are underlined; stop codon (in reverse primer) is in bold

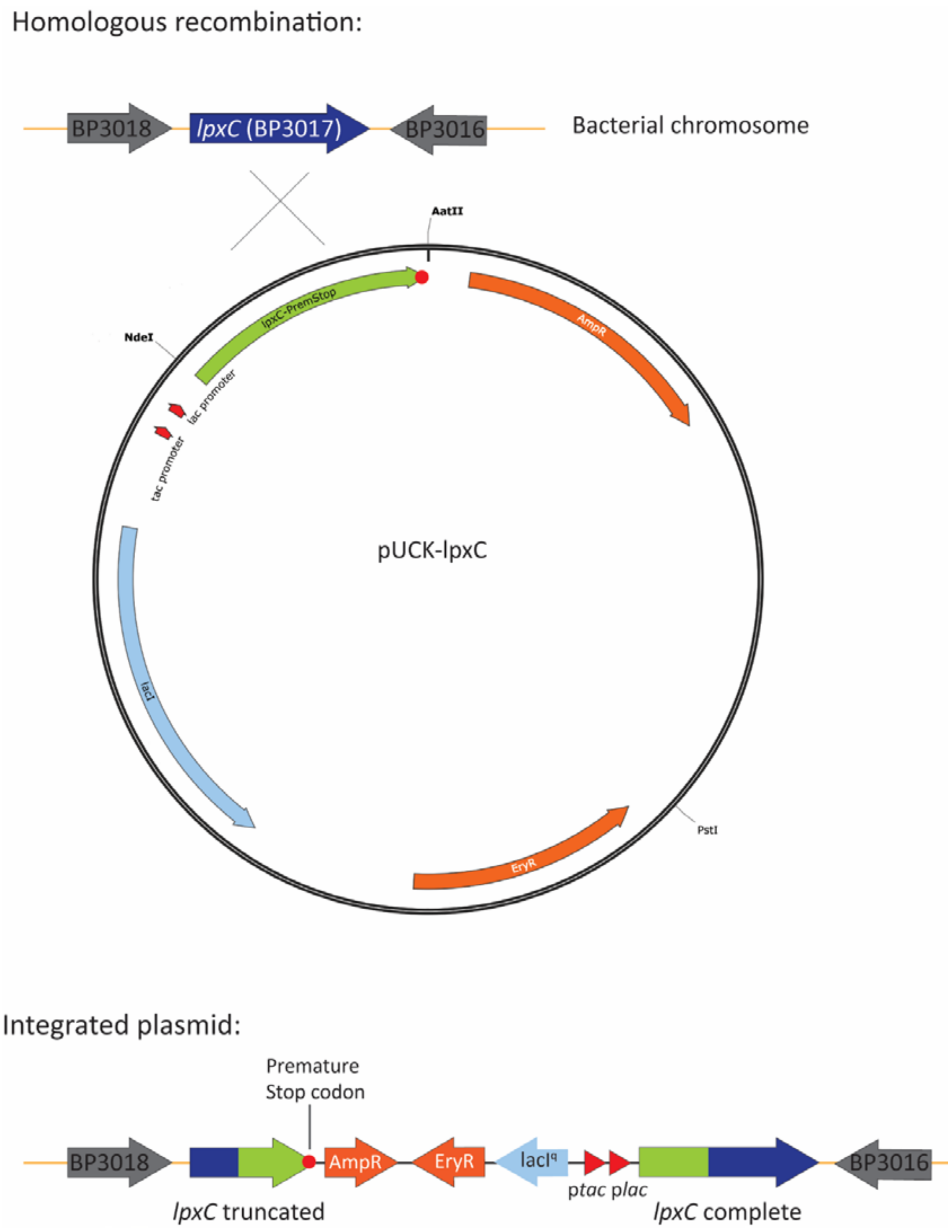

Figure S1 | Schematic representation of the integration of the pUCK-lpxC construct into the *B. pertussis* chromosome. Integration of the plasmid is achieved by homologous recombination

between a truncated *lpxC* gene (green) containing a premature stop codon (red circle) present on the plasmid and the intact chromosomal gene (dark blue). Chromosomal loci surrounding *lpxC* are depicted in grey. As a result of the integration, the intact *lpxC* copy with its own promoter is disrupted by the premature stop codon, whereas a new intact gene preceded by an IPTG-inducible double promoter (red arrowheads) is created. The presence of a *lacI*^{q} gene (light blue) allows for transcriptional repression in the absence of IPTG. The presence of antibiotic-resistance genes (orange) allows for selection of recombinants with the integrated plasmid. Restriction sites utilized during cloning are also depicted.

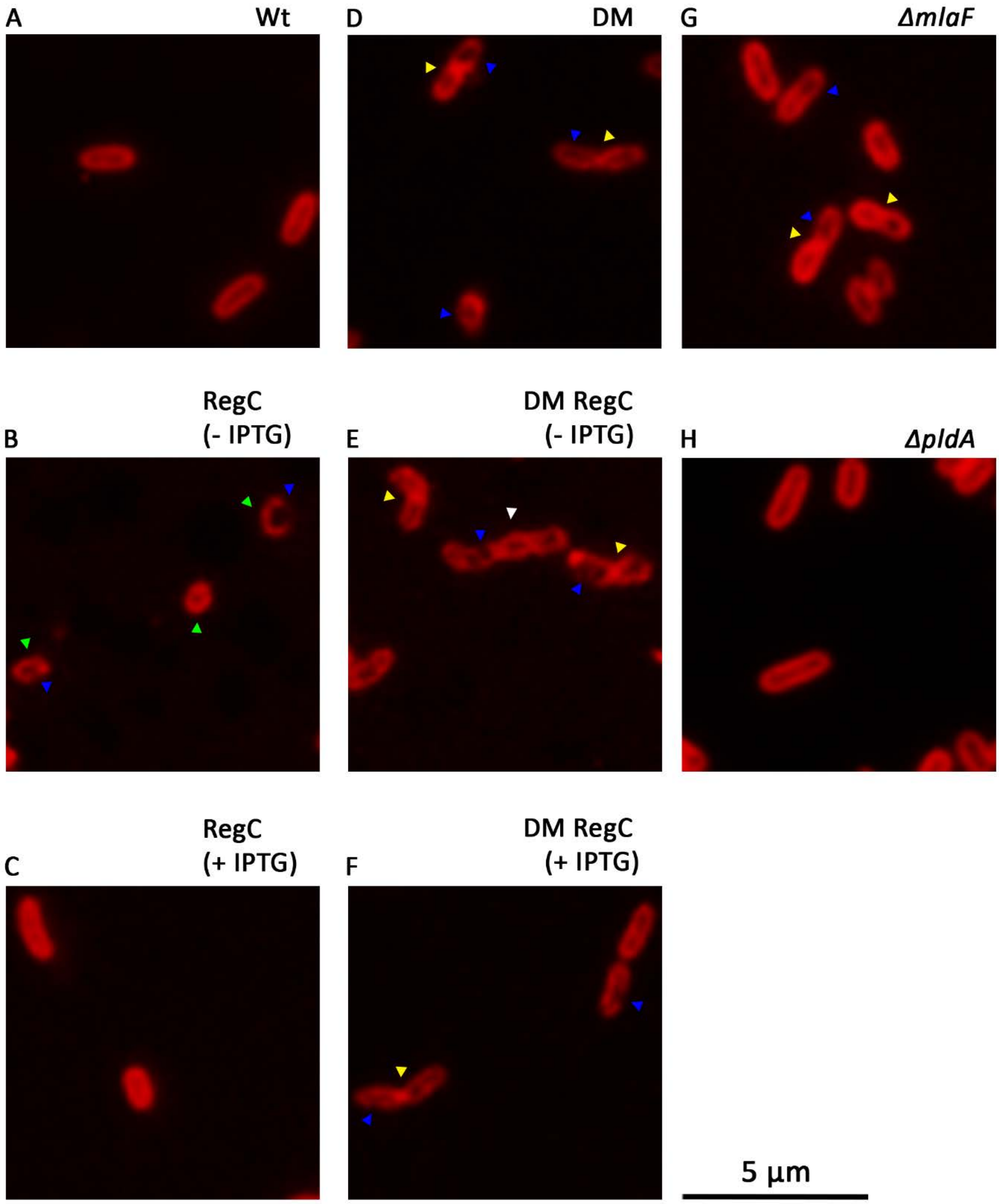

FIGURE S2 | Morphology of *B. pertussis* cells visualized by fluorescence microscopy. This figure shows magnified captions from **Figure 3**. Scale bar represents 5 µm. Examples of cells showing shorter and more rounded shape than the wild-type cells (green), uneven dye distribution (blue), or appearing in pairs (yellow) or short chains (white) are indicated with colored arrowheads.
